# Impact of repeat ablation of ventricular tachycardia in patients with structural heart disease

**DOI:** 10.1093/europace/euad367

**Published:** 2023-12-21

**Authors:** Joaquin Garcia Garcia, Arash Arya, Borislav Dinov, Andreas Bollmann, Rachel M A ter Bekke, Kevin Vernooy, Nikolaos Dagres, Gerhard Hindricks, Angeliki Darma

**Affiliations:** Department of Cardiac Electrophysiology, Heart Center of Leipzig, Struempellstrasse 39, Leipzig 04289, Germany; Department of Cardiac Electrophysiology, Halle University, Halle (Saale), Germany; Department of Cardiac Electrophysiology, Heart Center of Leipzig, Struempellstrasse 39, Leipzig 04289, Germany; Department of Cardiac Electrophysiology, Heart Center of Leipzig, Struempellstrasse 39, Leipzig 04289, Germany; Department of Cardiology, Cardiovascular Research Institute Maastricht (CARIM), Maastricht University Medical Centre (MUMC+), Maastricht, The Netherlands; Department of Cardiology, Cardiovascular Research Institute Maastricht (CARIM), Maastricht University Medical Centre (MUMC+), Maastricht, The Netherlands; Department of Cardiac Electrophysiology, Charité University Berlin, Berlin, Germany; Department of Cardiac Electrophysiology, Charité University Berlin, Berlin, Germany; Department of Cardiac Electrophysiology, Heart Center of Leipzig, Struempellstrasse 39, Leipzig 04289, Germany; Department of Cardiology, Cardiovascular Research Institute Maastricht (CARIM), Maastricht University Medical Centre (MUMC+), Maastricht, The Netherlands

**Keywords:** Repeat ablation in ventricular arrhythmias, Acute and long-term freedom from VT, Risks and complication management, Prognosis after repeat VT ablation

## Abstract

**Aims:**

Recurrences of ventricular tachycardia (VT) after initial catheter ablation is a significant clinical problem. In this study, we report the efficacy and risks of repeat VT ablation in patients with structural heart disease (SHD) in a tertiary single centre over a 7-year period.

**Methods and results:**

Two hundred ten consecutive patients referred for repeat VT ablation after previous ablation in our institution were included in the analysis (53% ischaemic cardiomyopathy, 91% males, median age 65 years, mean left ventricular ejection fraction 35%). After performing repeat ablation, the clinical VTs were acutely eliminated in 82% of the patients, but 46% of the cohort presented with VT recurrence during the 25-month follow-up. Repeat ablation led to a 73% reduction of shock burden in the first year and 61% reduction until the end of follow-up. Similarly, VT burden was reduced 55% in the first year and 36% until the end of the study. Fifty-two patients (25%) reached the combined endpoint of ventricular assist device implantation, heart transplantation, or death. Advanced New York Heart Association functional class, anteroseptal substrate, and periprocedural complication after repeat ablation were associated with worse prognosis independently of the type of cardiomyopathy.

**Conclusion:**

While complete freedom from VT after repeat ablation in SHD was difficult to achieve, ablation led to a significant reduction in VT and shock burden. Besides advanced heart failure characteristics, anteroseptal substrate and periprocedural complications predicted a worse outcome.

## Graphical Abstract

**Graphical Abstract:**
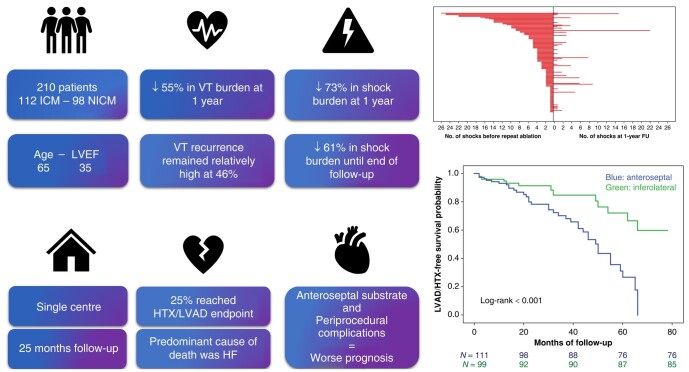

**What’s new?**

- Repeat ventricular tachycardia (VT) ablation in patients with structural heart disease led to a relevant reduction in shock and VT burdens.
- Outcomes regarding recurrence and mortality did not differ between patients with ischaemic and non-ischaemic cardiomyopathy in our cohort.
- Advanced New York Heart Association class, anteroseptal substrate, and periprocedural complications were associated with worse prognosis independently of the type of cardiomyopathy.
- One quarter of our cohort needed left ventricular assist device (LVAD)/heart transplantation (HTX) or died during follow-up. The main cause was deterioration of heart failure.

## Introduction

Over the past few decades, radiofrequency ablation (RFA) has been established as a reasonably effective and safe therapy for the management of ventricular arrhythmias (VA) in patients with structural heart disease (SHD). Several randomized controlled trials have shown RFA to be superior to antiarrhythmic drugs (AADs) regarding arrhythmia-free survival, reduction of spontaneous VA burden, and appropriate implantable cardiac defibrillator (ICD) therapies.^1–4^

Long-term recurrence after RFA varies greatly depending on the underlying SHD with reported rates ranging from 30–70%.^5–9^ A repeat procedure is often necessary, but these procedures tend to target worse tolerated VA, require more often epicardial access, and are longer and strenuous procedures.^10^ Acute success rates and long-term VA-free survival have been shown to be inferior in comparison with first RFA.^10,11^ In this study, we present data from a large tertiary centre over a 7-year period with the aim to analyse acute, long-term outcomes, and risks after repeat ablation for VA in a contemporary cohort of patients with SHD.

## Methods

### Study population

The present study was a retrospective series of 210 consecutive SHD patients who underwent repeat ablation for ventricular tachycardia (VT) at the Heart Center of Leipzig between January 2015 and December 2022. The full population of patients treated for VT during this time period was ∼658 patients. Patients with only one VT ablation procedure or patients who were previously ablated in other centres were excluded. The clinical decision to perform a repeat ablation was based on a combination of factors such as symptoms caused by the VT, haemodynamic worsening of the patient, shock and VT burdens, and contraindication or inefficiency of non-invasive approaches. In order to assess disease stage and facilitate the comparison of the cohort with those of other centres, we calculated the proposed I-VT score^12^ for recurrence and mortality before and after repeat ablation. Every patient gave written informed consent to the repeat ablation according to institutional guidelines. The study was performed in approval of the ethics committee.

Structural heart disease was classified into ischaemic (ICM) or non-ischaemic cardiomyopathy (NICM) according to a combination of medical history and findings from various imaging methods, biopsy, and electrophysiological studies. Baseline data of the study population reflected the status directly before the repeat ablation procedure. Values of left ventricular ejection fraction (LVEF), end-diastolic diameter (LVEDD), and volume (LVEDV) were retrieved from the closest transthoracic echocardiography to the repeat ablation. Electrical storm (ES) and incessant VT were determined according to the definitions present at the 2019 Heart Rhythm Society (HRS)/European Heart Rhythm Association (EHRA)/Asia Pacific Heart Rhythm Society (APHRS)/Latin America Heart Rhythm Society (LAHRS) expert consensus statement on catheter ablation of VA.^13^

### Electrophysiological study, epicardial access, and catheter ablation

The methodology of the ablation procedure has been described elsewhere.^14^ Put briefly, all patients underwent an electrophysiological study in the fasting state under deep sedation with propofol, midazolam, and fentanyl. When the VTs were not incessant, an induction protocol was performed using a stimulator (Biotronik Heart-Stimulator, Biotronik Worldwide, Berlin, DE). We used programmed electric stimulation from the right ventricular apex and outflow tract with four different drive cycle lengths (500, 430, 370, and 330 ms) and introduction of up to three extrastimuli until a ventricular effective refractory period or a coupling interval of 200 ms was reached. If not inducible, additional stimulation in the left ventricle was performed. The same induction protocol was used to re-induce the VT after the ablation. We used fluoroscopy-guided transseptal puncture to access the left atrium, and a long sheath (Agilis-L Abbott, St. Paul, MN, USA) was introduced into the LA.

The decision for an epicardial approach was based on the electrocardiogram (ECG) characteristics of the VT, the evidence of epicardial substrate [late gadolinium enhancement (LGE) in cardiovascular magnetic resonance (CMR), unipolar map], and the unsuccessful endocardial elimination of the VT and the haemodynamic stability of the patient during the procedure. The pericardium was accessed percutaneously using the Sosa *et al.* technique.^15^ The epicardial puncture through the subxiphoid window was done using a long sheath (Agilis EPI Abbott, St. Paul, MN, USA). Haemodynamic devices were implemented at the discretion of the physician and preferred in patients with ejection fraction <20%.

Electroanatomic mapping was performed using the CARTO-3 system (Biosense Webster, Diamond Bar, CA, USA) or the Precision EnSite Navigation system (Abbott, St. Paul, MN, USA). Areas of low voltage (<1.5 mV), unexcitable scar (<0.5 mV and loss of capture when pacing), late potentials, and fragmented potentials were identified. Due to the haemodynamic instability of the majority of induced VTs, pace mapping was performed, areas of long S-QRS delays and pace mapping–matched QRS morphology of an induced VT targeted. The substrate categorization in anteroseptal or inferolateral was decided after reviewing the electrophysiological study findings, the VT morphologies, and if available (in 70 patients), the presence of LGE in CMR imaging.

Radiofrequency alternating current was delivered in a unipolar mode between the irrigated tip electrode of the ablation catheter (SmartTouch or F-Type, irrigated tip, Thermocool, Biosense Webster, Diamond Bar, CA, USA; Therapy™ Flexability™ Ablation Catheter, Abbott, St. Paul, MN, USA) and an external neutral electrode. The standard ablation setting consisted of an upper temperature limit of 42°C, a power of 40–50 W, and a flow rate of up to 30 mL/min of normal saline. Half-normal saline, dextrose 5% in water, and sequential unipolar or bipolar ablation were not used in any instance. The ablation was terminated if the clinical VT was ablated, no more VTs were induced, or the patient deteriorated haemodynamically. If a pericardial access was gained, the pericardial sheath was removed at the end of the ablation or was replaced with a soft catheter to be removed maximum after 24 h in the absence of bleeding.

### Outcomes

Long-term outcomes included the following: (i) survival free of the composite of death, implantation of left ventricular assist device (LVAD), or cardiac transplantation (HTX); (ii) presence of VT recurrence, defined as presence of symptomatic VT or need for shock or ATPs or slow VTs which granted a change in therapeutic approach; and (iii) in case of VT recurrence, evaluation of sustained VT and shock burden post ablation (ICD shock or external cardioversion/defibrillation). All VT recurrencies, including in-hospital, were taken into account. As a result, no blanking period after the ablation procedure was established. Acute procedural outcomes consisted of non-inducibility of the clinical VT and of any inducible VT. The device programming after the procedure typically included a VT zone able to detect the slowest clinical and/or induced VT. If additional AADs were initiated, this was considered when programming the tachycardia therapy zones.

### Complications

Periprocedural complications were classified depending on the casual relationship to the procedure or the hospitalization. Complications directly related to the repeat ablation procedure were further grouped into two categories according to severity. Major (life-threatening) complications included stroke, transient ischaemic attack (TIA), acute pericardial tamponade, and vascular injuries needing transfusion. Minor complications involved arteriovenous (AV) fistula and occlusion of the superficial femoral artery (AFS) treated conservatively as well as reactive pericardial effusion without indication for drainage.

### Follow-up

Follow-up was defined from the time of repeat ablation to the time of last follow-up or death. Documentation of VT was achieved by inspection of the ICD storage and in cases of below detection VT or lack of device by 12-lead ECG or telemonitoring. Follow-up included review of records of all hospital and outpatient clinic visits and discussion with referring cardiologists and primary care physicians. Shock burden considered not only ICD but also external shocks. Antiarrhythmic drugs would typically be discontinued if the ablation was successful and the recurrence risk was considered as low. In case of recurrence in the first weeks after the ablation or in the presence of large substrate with multiple VTs and high recurrence probability or unsuccessful ablation, patients were treated with additional AADs, re-ablation, or escalation of the heart failure management.

### Statistical analysis

Continuous variables were reported as mean ± standard deviation and categorical variables as frequencies. Continuous variables were compared using Student’s *t*-test, while categorical variables were compared using the *χ*^2^ test. In the patients with multiple ablations, the baseline clinical characteristics and procedural data of the first repeat procedure were included. Univariable and multivariable cox regression analyses were performed in order to determine the predictive factors. The variables in the univariable analysis were then included in the multivariate regression analysis for the determination of hazard ratio (HR) and its 95% confidence interval (CI). A *P*-value of ≤0.05 was considered to be statistically significant. The variables were considered normally distributed if ∼68% of the value was within 1 SD from the mean. Otherwise, they were considered as non-normally distributed and reported in *Table 1* through median values. All analyses were performed using SPSS v24.0 (SPSS Inc., Chicago, IL, USA).

**Table 1:** Baseline and periprocedural characteristics

Variable	Total	ICM	NICM	*P*-value
	(*n* = 210)	(*n* = 112)	(*n* = 98)	
Baseline characteristics				
Male	191	106	85	0.046
Female	19	6	13	
Age [median years, (IQR)]	65 (58–72)	66 (59–74)	64 (55–70)	0.014
NYHA functional class				0.181
I–II	127	63	64	
III–IV	83	49	34	
Art. hypertension	181	108	73	<0.001
Diabetes mellitus	80	51	29	0.013
Renal dysfunction	145	83	62	0.090
COPD	22	15	7	0.140
Atrial fibrillation				0.566
Paroxysmal	61	36	25	
Persistent	56	29	27	
LVEF (mean % ± SD)	35.4 ± 13	31.7 ± 11	39.7 ± 13	<0.001
LVEDV (mean mL ± SD)	203.5 ± 83	224.7 ± 84	179.7 ± 75	<0.001
LVEDD (mean mm ± SD)	60.9 ± 10	62.5 ± 9	59.0 ± 10	0.009
BMI (mean ± SD)	28.9 ± 5	29.3 ± 5	28.5 ± 5	0.253
ICD				0.106
One-chamber	67	38	29	
Two-chamber	54	26	28	
Three-chamber	79	46	33	
Antiarrhythmic medication (baseline)	119	57	62	0.071
Electrical storm	128	72	56	0.290
Incessant VT	32	17	15	0.980
VT burden [median episodes at baseline, (IQR)] * incessant VT excluded	8 (2–25)	7.5 (2–23)	9 (2–28)	0.972
Shock burden [median episodes at baseline (IQR)]	1 (0–4)	1 (0–4)	1 (0–4)	0.900
Procedural data and outcome				
Number of mmVTs				0.738
1–3	160	86	74	
>3	49	25	24	
Anteroseptal vs.	112	57	55	0.542
inferolateral substrate	98	54	44	
Epicardial ablation	49	7	42	<0.001
Non-inducibility	150	86	64	0.308
Clinical VT inducible	9	5	4	
Non-clinical VT ind.	23	10	13	
No inducible overall	15	7	8	
No test at proc. end	13	4	9	
Complication	28	11	17	0.097
Antiarrhythmic medication (follow-up)	147	74	73	0.281
Recurrence	97	50	47	0.631
VT burden post (median no. of episodes when recurrence (IQR)]				
First year	7 (1–36)	4 (0–19)	13 (3–47)	0.048
End follow-up	15 (3–42)	12 (2–27)	21 (5–54)	0.201
ICD shock burden post [median no. of episodes when recurrence, (IQR)]				
1st year	0 (0–2)	0 (0–1)	1 (0–2)	0.103
End follow-up	1 (0–3)	1 (0–2)	1 (0–4)	0.128
Third ablation	0.90 ± 1.4	0.87 ± 1.4	0.94 ± 1.4	0.704
Combined endpoint	52	29	23	0.685
LVAD	18	11	7	0.489
HTX	10	4	6	0.386
Death	36	19	17	0.941

BMI, body mass index; COPD, chronic obstructive pulmonary disease; HTX, heart transplantation; ICD, implantable cardiac defibrillator; ICM, ischaemic cardiomyopathy; IQR, interquartile range; LVEDD, left ventricle diastolic diameter; LVEDV, left ventricle end-diastolic volume; LVEF, left ventricular ejection fraction; LVAD, left ventricular assist device; NICM, non-ischaemic cardiomyopathy; NYHA, New York Heart Association; SD, standard deviation; VT, ventricular tachycardia.

## Results

### Baseline and procedural characteristics

The baseline features of the study population are summarized in *Table 1*. Compared with the ICM group (*n* = 112), NICM patients (*n* = 98) were more often female and younger and had fewer comorbidities, such as hypertension and diabetes. Non-ischaemic cardiomyopathy patients had also a more preserved LVEF and smaller LVEDV and LVEDD. No significant differences were observed in the proportion of patients on AADs and burden of VT episodes or shocks before the repeat ablation.

Regarding procedural characteristics, the NICM group underwent significantly more epicardial ablations. There were no significant differences in number of induced VT morphologies, substrate localization, inducibility at the end of the procedure, or the use of AADs after the procedure. The I-VT score of the cohort for recurrence was 1.25 before and 0.8 after repeat ablation. The I-VT score for mortality before repeat ablation was 0.45 and 0.44 after the procedure.

### Indication for repeat ablation

The indication to perform a repeat ablation was electrical storm/incessant VT in 160 of 210 patients (76%), slow/below detection VTs in 18 cases (9%), 1–5 VT episodes in 21 patients (10%), and more than 5 episodes in 11 patients (5%). Of the patients with 1–5 VT episodes, multiple ICD shocks were recorded in 24% of the cases, a single ICD shock in 29%, and repeated ATP therapies in 47%. Mean time from initial to repeat ablation was 19 months in patients with electrical storm/incessant VT, 16 in those with slow/below detection VT as indication for repeat ablation, 17 in patients with 1–5 VT episodes, and 37 for patients with more than 5 VT episodes.

After comparing the cycle length of the induced VT and 12-lead ECG (when available) of the repeat ablation with those of the initial procedure, a definite recurrence of a previously ablated or induced VT was documented in 20 of the 210 patients (10%). The indication to repeat ablation in the 20 patients with definite recurrence was electrical storm/incessant VT in 12 patients, 1–5 VT episodes in 5 patients, more than 5 VT episodes in 1 case, and slow/below detection VT in the remaining 2 patients. In 102 patients, new VT morphologies were present (49%). Thirty-three (16%) patients presented with large substrates and multiple VT morphologies in both procedures making a direct comparison difficult. Twenty-seven (13%) patients presented with multiple VTs during the first procedure and had recurrence of one induced VT morphology leading to the repeat procedure (some of the VTs not effectively ablated, some not ablated because of deteriorating haemodynamic/respiratory situation, need for epicardial access, etc.). In the rest of the cohort, no meaningful distinction was possible.

Progression of substrate (defined as documentation of new areas of low voltage and/or late potentials) from the initial to the repeat ablation was identified in 68 of the 210 patients (32%), of which 28 (41%) had ICM and 40 (59%) had NICM. Mean time to repeat ablation was 28 months in the cohort of patients with progressive substrate, considerably longer in comparison with patients without definite progression of the substrate (15 months). Patients with ICM and progressive substrate had a longer time span between ablations (33 months) in contrast to patients with NICM (25 months). Of the 40 patients with NICM, 10 had post-myocarditis (25%), 6 had familial DCM (15%), and 13 had not further classified NICM (32%). Sarcoidosis, ARVC/ALVC, hypertensive cardiomyopathy, and valvulopathy were present in two patients each. The rest of the patients had various other cardiomyopathies (8%).

### Periprocedural complications

A total of 28 patients (13%) developed complications. Fourteen of those suffered procedure-related complications (8 major, 6 minor), while the other 14 experienced hospitalization-related complications (*Table 2*). In four patients, cardiac tamponade occurred during the procedure, requiring emergency thoracotomy in two patients, while in the other two cases, the bleeding stopped after pericardial drainage and administration of protamine. One patient suffered a stroke in the first days after the ablation, and another had a transient ischaemic event without further sequelae. Two patients needed blood transfusion due to vascular complications. Four patients presented with pericardial effusion with pericarditis symptoms and were treated conservatively. Arterial vascular access led to the occlusion of an AFS and to an AV fistula, which were asymptomatic and were also treated conservatively. Ten patients suffered nosocomial pneumonia or prolonged respiratory distress, and three patients needed dialysis. Last, one patient suffered a device endocarditis due to bacteraemia and underwent ICD explantation. The complications occurring during the repeat ablation were compared with complications occurring during the first ablation in the same cohort. While no significant changes in the interventional part were documented, the cohort showed significantly more hospitalization-associated complications during the second ablation. Possible explanations to this could include increased complexity and duration of the ablation procedure, age and disease progression, and increased frailty.

**Table 2:** Periprocedural major and minor complications

Procedure-associated complications	
Major	8
Acute tamponade during ablation	4
Peri-interventional stroke/TIA	2
Vascular complication with transfusion	2
Minor	6
Pericardial effusion without puncture	4
AFS occlusion	1
AV fistula	1
Hospitalization-associated complications	14
Nosocomial pneumonia/respiratory distress	10
Prolonged cardiorenal decompensation with need for dialysis	3
Device endocarditis	1

AFS, superficial femoral artery; AV, arteriovenous; TIA, transient ischaemic attack.

### Long-term outcome

**Figure 1:**
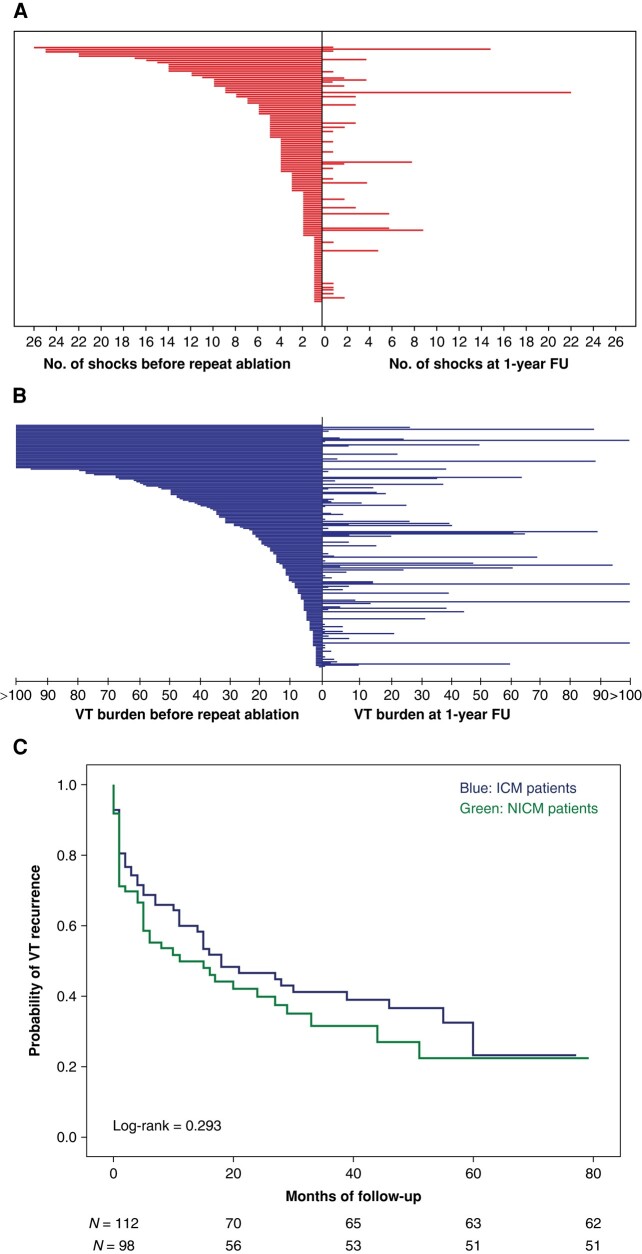
(*A*) Illustration of shock burden of the cohort at baseline (left, only episodes up to 1 year before repeat ablation considered) and during the first year post repeat ablation (right). FU, follow-up. (*B*) Graphic of VT burden at baseline (left, only episodes up to 1 year before repeat ablation considered) and during the first year post repeat ablation (right, *patients admitted in incessant VT excluded). FU, follow-up. (*C*) Impact of cardiomyopathy on VT recurrence (blue line, ICM patients; green, NICM, *n* = number of patients).

**Figure 2:**
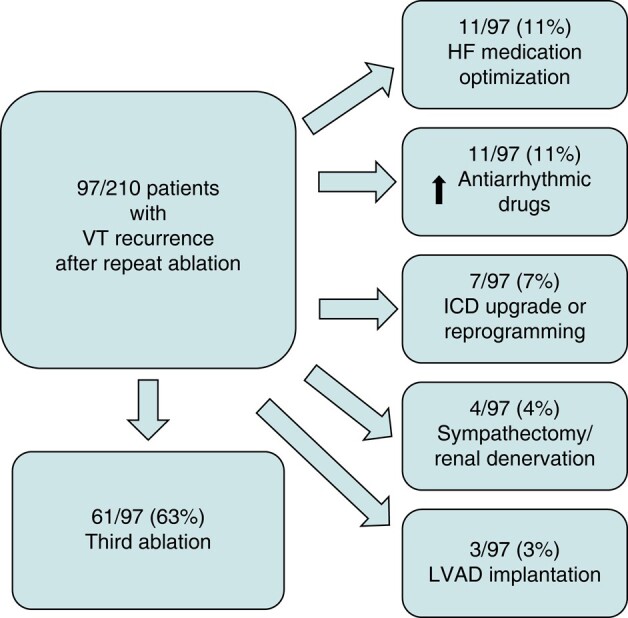
Flowchart of VT recurrence management in SHD patients post repeat ablation. HF, heart failure; ICD, internal cardioverter defibrillator; LVAD, left ventricular assist device.

Mean follow-up duration after the repeat VT ablation was 25 ± 20 months. Ventricular tachycardia recurrence was documented in 97 patients (46%) until the end of follow-up. Repeat ablation led to a 73% reduction of shock burden in the first year and a 61% reduction until the end of follow-up (*Figure 1A*). Regarding VT burden, repeat ablation led to a reduction of 55% in the first year and 36% until the end of follow-up (*Figure 1B*). This reduction of VT burden is however underestimated, since the 32 patients presenting in incessant VT before the repeat ablation could not be adequately represented and were thus not included in the VT burden analysis. The NICM group had a higher VT burden recurrence rate in the first year of follow-up. However, the cumulative VT recurrence until the end of follow-up was similar between ICM and NICM patients (*Figure 1C*). Of the 97 patients with recurrence, 61 underwent a third ablation during the follow-up, 11 patients were treated conservatively with optimization of heart failure medication and electrolyte correction, while in 11 cases, AADs were escalated. Furthermore, seven patients underwent an ICD upgrade or re-programming, four had bilateral sympathectomy or renal denervation, and three underwent LVAD implantation (*Figure 2*). For patients receiving multiple therapies, these were prioritized as following: LVAD implantation > third ablation > sympathectomy/renal denervation > ICD upgrade > increase on antiarrhythmics > heart failure medication optimization and ICD re-programming.

The combined endpoint was reached in 52 patients; overall, 18 patients underwent LVAD implantation, 10 received a heart transplant, and 36 patients died. In a total of 12 instances, recurrent VA was the driving cause of HTX/LVAD implantation. No differences regarding recurrence or LVAD/HTX-free survival were reported in both groups (see Supplementary material online, *Figure S1A*). Predominant cause of death was terminal heart failure or cardiogenic shock (11 patients, 1 after LVAD deactivation, 1 due to recurrent VT), followed by sepsis (8 patients), while in the remaining 17 patients, the exact cause could not be reported.

Regarding the 61 patients (54 men, median age 65 years) who underwent a third ablation in order to manage the VT recurrence, the mean LVEF was 37 ± 12%, and mean LVEDV and mean LVEDD were 187 ± 74 mL and 60 ± 10 mm, respectively. Thirty-two (52%) of these patients had ICM, and 39 (64%) had achieved non-inducibility after the second ablation. In four cases (7%), the clinical VT remained inducible at the end of the first repeat procedure. As for the rest, no test was performed at the end of the intervention in seven patients (11%), a non-clinical VT remained inducible in eight (13%), and no VT was inducible at the beginning or end of the procedure in three cases (5%).

### Predictors of ventricular tachycardia recurrence and left ventricular assist device/heart transplantation–free survival

**Figure 3:**
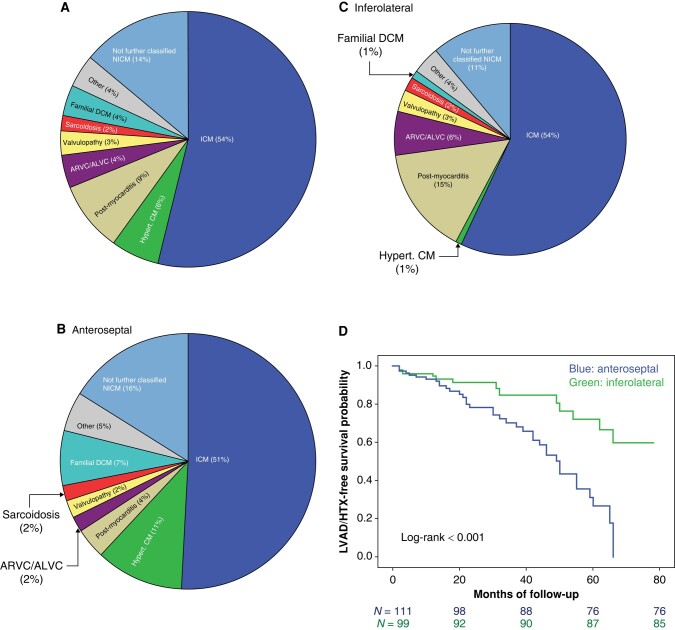
Pie graphic on cardiomyopathy classification of the cohort (*A*), in patients with anteroseptal substrate (*B*), and inferolateral substrate (*C*) with corresponding Kaplan–Meier curve depicting the impact of anteroseptal substrate on LVAD/HTX-free survival (*D*). Blue, anteroseptal group; green, inferolateral, *n* = number of patients.

The results of the univariate and multivariable Cox proportional hazards analyses to determine the association between baseline covariates and outcome events are reported in the Supplementary material online, *Tables S1* and *S2*. On multivariable analysis, the use of AADs at the end of follow-up (HR: 3.7; 95% CI: 2.1–6.8; *P* < 0.001), need for epicardial ablation (HR: 1.7; 95% CI: 1.1–2.7, *P* = 0.015), and renal dysfunction (HR: 1.6; 95% CI: 1.1–2.6, *P* = 0.036) was independently associated with VT recurrence during follow-up. Independent predictors of death/LVAD or HTX during follow-up were the presence of anteroseptal substrate (HR: 3.0; 95% CI: 1.5–6.3; *P* = 0.002, *Figure 3*), periprocedural complication (HR: 2.0; 95% CI: 1.0–4.1; *P* = 0.046), and advanced New York Heart Association (NYHA) functional class (HR: 2.0; 95% CI: 1.0–3.9; *P* = 0.044).

## Discussion

The main findings from this study are as follows: (i) the recurrence rate after repeat VT ablation in SHD patients remained relatively high with 46%. (ii) Repeat VT ablation in patients with SHD led to a relevant reduction in shock and VT burdens. (iii) Twenty-five per cent of the cohort reached the combined endpoint of LVAD/HTX or death after a 25-month follow-up. (iv) Renal dysfunction, need for epicardial access, and use of AADs during follow-up were independent predictors of recurrence. (v) Advanced NYHA class, anteroseptal substrate, and periprocedural complications were associated with worse prognosis independently of the type of cardiomyopathy. (vi) Dominant cause of death was terminal heart failure.

In this study, the efficacy and risk of repeat ablation in SHD patients were analysed. We found that repeat ablation led to a significant reduction of shock and VT burden in this sick population, which can have a substantial impact in need for hospitalization, quality of life, and possibly mortality. These results are in line with previous studies on repeat VT ablation, which also demonstrated higher recurrence rates in comparison with first ablations.^10,11^ Surprisingly, the outcomes regarding recurrence and mortality did not differ between ICM and NICM patients in our cohort. However, these were highly selected cases referred to a tertiary reference centre for VT ablation; thus, selection bias and substrate particularities in ICM patients are to be expected.

**Figure 4:**
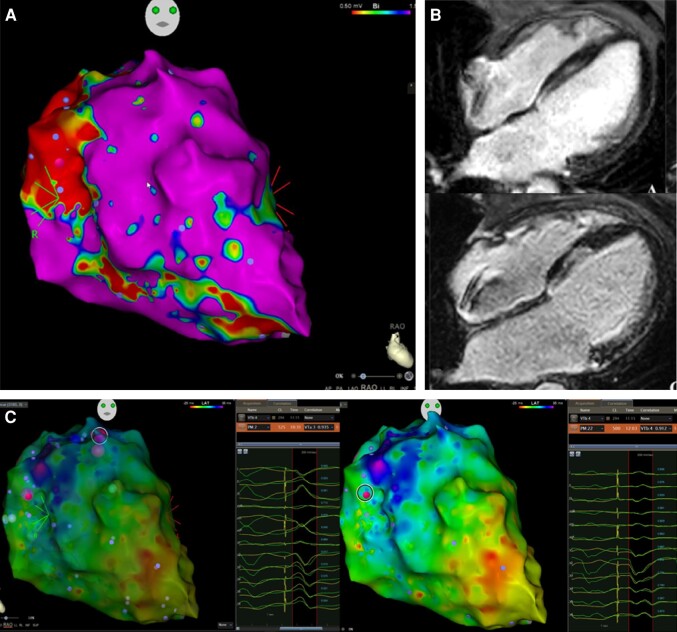
Example of NICM patient of the cohort with septal substrate on the voltage map (*A*), with intramural LGE evidence in CMR (*B*) and corresponding anterosuperior and anteroseptal VT exits at the margin of late activation area in sinus rhythm endocardial (illustrated with CARTO-3 system). Both VT morphologies were clinical VTs. The activation map in sinus rhythm (*C*) demonstrates the areas of late activation at the basal anteroseptal area, where the VT exits were identified. After extensive substrate modification of the late activation area, no VTs were inducible at the end of the procedure.

In our study, anteroseptal substrate was the strongest predictor for worse long-term outcome. This is also in agreement with previous publications on VT ablation and LGE localization.^16–20^ As demonstrated in *Figure 3*, the substrate distribution differed depending on the patients’ cardiomyopathy type; nevertheless, anteroseptal substrate was generally more often present in NICM patients. Anteroseptal substrate has been associated with intramural myocardial re-entries, involvement of diseased His-Purkinje fibres, fast unmappable VTs, and malignant premature ventricular contractions initiating ventricular fibrillation (*Figure 4*). All of the above contribute to reduced success rates in acute and long-term ablation results, to increased need for bradycardia pacing, and to a rapid progression of congestive heart failure, all leading to a decrease in survival. With increasing expansion of indication of VT ablation in NICM patients, this has become a problem in clinical practice. The major limitation for anteroseptal substrate seems to be the depth of the ablation lesions. New technologies, if established widely in the future, such as pulsed field ablation (PFA), cryoablation in the ventricle, or radiotherapy, could help achieve better outcomes. However, for the time being, patients with anteroseptal substrates appear to need closer follow-up (ideally with telemonitoring and HF nurses), more antiarrhythmics, and eventually quicker referral to LVAD/HTX centres.

One quarter of our cohort needed LVAD/HTX or died during follow-up. The main cause of death or HTX/LVAD implantation was deterioration of heart failure. In our study, patients who suffered procedure or hospitalization-related complications had a two-fold increased risk of significant clinical deterioration (see Supplementary material online, *Figure S1B*). This underlines how fragile these patients are and why the management of SHD patients with VT does not end with ablating the VT. Establishing intensive care–specialized VT units and working in close cooperation with electrophysiologists, intensive care physicians, heart failure specialists, radiologists, and geneticists is of pivotal importance and can make the difference in the prognosis of these patients.

### Reasons for failure of ventricular tachycardia ablation

The main reasons for recurrence are usually incomplete substrate identification, ineffective ablation lesions, or disease progression. The differences on substrate analysis between the procedures in our cohort depended on the type of cardiomyopathy, the map detail according to different mapping catheters/systems, and the amount of ablation performed in the initial procedure. In our study, NICM patients presented more often with progressive substrates. Moreover, the time span for repeat ablation in NICM patients was significantly shorter compared with ICM patients.

Understanding the arrhythmogenic mechanism seems crucial to ablation success in these patients. Especially in NICM, complex scar architecture extending from the endocardial to the epicardial layers of the myocardium is often present. This renders multiple chamber high-density mapping essential, in order to identify the critical isthmi.^21–23^ Similar to these NICM particularities, contemporary ICM patients often present with ‘patchy’ lesions as a result of quicker revascularization, which are much more challenging than the typical compact endocardial infarction scar. Since the introduction of high-density mapping with multipolar catheters, the 3D mapping systems can provide more accurate visualization of the re-entry circuits, thus enabling more precise and effective ablation lesions.^21,24,25^ Furthermore, the rising pre-interventional imaging with identification of areas of interest using the available high-resolution computed tomography (CT) or CMR images and image integration in electroanatomic maps could also provide valuable substrate information.^22^ These new technologies, when combined with new sources of energy to improve the ablation lesion depth (PFA, needle RFA, cryoablation, radioablation), could improve the long-term outcomes of these procedures.^26–32^

### Limitations

The current study summarizes a retrospective, non-randomized experience from a single tertiary referral centre, so that potential selection and referring bias must be considered. The cohort consisted of highly selected patients with a variety of diagnoses and underlying arrhythmia substrates. Subsequently, the ablation outcome may differ significantly. The centre participating in this study has a relatively high volume of patients referred for repeat ablations compared with other centres, which may limit the generalizability of our results. A time frame of 7 years indicates that the technology regarding ablation as well as the strategy of VT mapping evolved significantly which may also have an impact on the results. A direct comparison of the total scar area and areas of LP between the initial and the repeat procedure was due to heterogeneous conditions (different operators, mapping systems, bipolar/multipolar catheters, indication emergency, haemodynamic/respiratory situation, different mapping times) not possible.

## Conclusions

Repeat VT ablation in SHD patients led to a 73% reduction of shock burden in the first year and a 61% reduction until the end of follow-up. Similarly, VT burden was reduced to 55% and 36%, respectively. Apart from advanced heart failure characteristics, anteroseptal substrate and periprocedural complications predicted a worse outcome.

## Supplementary Material

euad367_Supplementary_Data

## Data Availability

The data that support the findings of this study are available from the corresponding author upon reasonable request.
