# Supplementary material for: Impact of repeat ablation of ventricular tachycardia in patients with structural heart disease
Source: Europace. 2023 Dec 21;26(1):euad367. doi: 10.1093/europace/euad367 (PMC10755192; doi:10.1093/europace/euad367)
Supplement: euad367_Supplementary_Data [file euad367_supplementary_data.docx]

**Supplementary material**

Table 1. Univariable and multivariable analysis regarding VT-recurrence.

| **Variable** | **Total** | **No recurrence** | **Recurrence** | **p-Value**  **univariate** | **p-Value**  **multivariate** |
| --- | --- | --- | --- | --- | --- |
| **Baseline characteristics** |  |  |  |  |  |
| Male  Female | 191  19 | 104  9 | 87  10 | 0.555 | 0.002; HR 0.3  CI 0.2-0.7 |
| Age (median years, [IQR]) | 65 [58-72] | 65 [59-73] | 65 [56-72] | 0.356 | 0.212 |
| ICM  NICM | 112  98 | 62  51 | 50  47 | 0.631 | 0.722 |
| NYHA functional class   - I-II - III-IV | 127  83 | 71  42 | 56  41 | 0.451 | 0.267 |
| Art. hypertension | 181 | 97 | 84 | 0.874 | 0.553 |
| Diabetes mellitus | 80 | 44 | 36 | 0.709 | 0.624 |
| Renal dysfunction | 145 | 77 | 68 | 0.709 | 0.036; HR 1.6  CI 1.1-2.6 |
| COPD | 22 | 17 | 5 | 0.020 | 0.624 |
| Atrial fibrillation   - paroxysmal - persistent | 61  56 | 32  31 | 29  25 | 0.952 | 0.810 |
| LV-EF (mean % ± SD) | 35.4±13 | 35.2±13 | 35.7±12 | 0.794 | 0.649 |
| LV-EDV (mean ml ± SD) | 203.5±83 | 204.5±82 | 202.4±84 | 0.858 | 0.962 |
| LV-EDD (mean mm ± SD) | 60.9±10 | 60.7±9 | 61.0±10 | 0.781 | 0.583 |
| BMI | 28.9±5 | 28.3±5 | 29.6±6 | 0.047 | 0.712 |
| ICD   - 1-ch - 2-ch - 3-ch | 67  54  79 | 32  32  42 | 35  22  37 | 0.440 | 0.660 |
| Antiarrhythmic medication (baseline) | 119 | 58 | 61 | 0.092 | 0.923 |
| Electrical storm | 128 | 66 | 62 | 0.414 | 0.256 |
| Incessant VT | 32 | 21 | 11 | 0.145 | 0.579 |
| **Procedural data and outcome** |  |  |  |  |  |
| Number of mmVTs  1-3  >3 | 160  49 | 94  19 | 66  30 | 0.014 | 0.195 |
| Epicardial ablation | 49 | 18 | 31 | 0.006 | 0.015; HR 1.7  CI 1.1-2.7 |
| Non-inducibility  Inducible VTs  - clinical VT  - non-clinical VT  No inducible overall  No test end | 150  9  23  15  13 | 87  1  13  7  5 | 63  8  10  8  8 | 0.055 | 0.652 |
| Anteroseptal substrate  Inferolateral substrate | 111  99 | 56  57 | 55  42 | 0.301 | 0.836 |
| Complication | 28 | 13 | 15 | 0.400 | 0.501 |
| Antiarrhythmic medication (follow-up) | 146 | 63 | 83 | <0.001 | <0.001; HR 3.7  CI 2.1-6.8 |

Table 2. Univariable and multivariable analysis of combined endpoint (death, LVAD implantation or HTX).

| **Variable** | **Total** | **No combined endpoint** | **Combined endpoint** | **p-Value**  **univariate** | **p-Value**  **multivariate** |
| --- | --- | --- | --- | --- | --- |
| **Baseline characteristics** |  |  |  |  |  |
| Male  Female | 191  19 | 145  13 | 46  6 | 0.470 | 0.833 |
| Age (median years, [IQR]) | 65 [58-72] | 64 [58-73] | 67 [59-72] | 0.487 | 0.710 |
| ICM  NICM | 112  98 | 83  75 | 29  23 | 0.685 | 0.335 |
| NYHA functional class  I-II  III-IV | 127  83 | 107  51 | 20  32 | <0.001 | 0.044; HR 2.0  CI 1.0-3.9 |
| Art. hypertension | 181 | 135 | 46 | 0.584 | 0.569 |
| Diabetes mellitus | 80 | 56 | 24 | 0.188 | 0.380 |
| Renal dysfunction | 145 | 103 | 42 | 0.035 | 0.605 |
| COPD | 22 | 17 | 5 | 0.815 | 0.422 |
| Atrial fibrillation  paroxysmal  persistent | 61  56 | 42  40 | 19  16 | 0.145 | 0.500 |
| LV-EF (mean % ±SD) | 35.4±13 | 37.7±13 | 28.7±9 | <0.001 | 0.005; HR 0.95  CI 0.9-1.0 |
| LV-EDV (mean ml ±SD) | 203.5±83 | 195.5±78 | 230.1±93 | 0.011 | 0.885 |
| LV-EDD (mean mm ±SD) | 60.9±10 | 60.3±10 | 62.3±10 | 0.121 | 0.048; HR 0.95  CI 0.9-1.0 |
| BMI | 28.9±5 | 28.9±5 | 28.9±5 | 0.987 | 0.919 |
| ICD  1-chamber  2-chamber  3-chamber | 67  54  79 | 55  47  48 | 12  7  31 | 0.002 | 0.633 |
| Antiarrhythmic medication (baseline) | 119 | 88 | 31 | 0.621 | 0.634 |
| Electrical storm | 128 | 92 | 36 | 0.158 | 0.342 |
| Incessant VT | 32 | 24 | 8 | 0.973 | 0.836 |
| **Procedural data and outcome** |  |  |  |  |  |
| Number of mmVTs  1-3  >3 | 160  49 | 122  36 | 38  13 | 0.692 | 0.541 |
| Epicardial ablation | 49 | 38 | 11 | 0.668 | 0.878 |
| Non-inducibility  - clinical VT inducible  - non-clinical VT induc.  No inducible overall  No test end of proc. | 150  9  23  15  13 | 119  5  19  10  5 | 31  4  4  5  8 | 0.008 | 0.878 |
| Anteroseptal substrate  Inferolateral substrate | 111  99 | 73  85 | 38  14 | 0.001 | 0.002; HR 3.0  CI 1.5-6.3 |
| Complication | 28 | 15 | 13 | 0.004 | 0.046; HR 2.0  CI 1.0-4.1 |
| Antiarrhythmic medication (follow-up) | 147 | 104 | 43 | 0.021 | 0.156 |
| Recurrence | 97 | 61 | 36 | <0.001 | 0.210 |

Figure 1

1. Impact of cardiomyopathy on LVAD/HTX-free survival (blue line: ICM patients, green: NICM, N= number of patients).


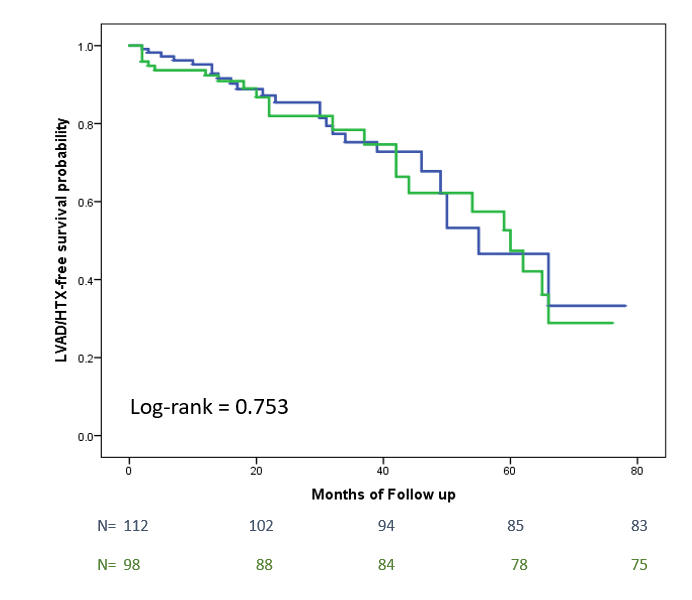


1. Impact of occurrence of procedure-related or hospitalisation-related complication on LVAD/HTX-free survival (blue line: without any complication, green: with complication, N= number of patients).


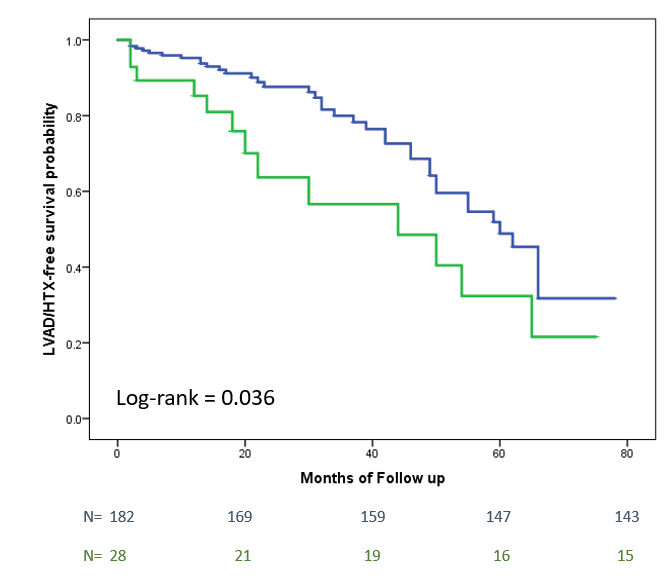


Figure 2a-d. Life tables analysis for recurrence (a-b) and LVAD/HTX-free survival (c-d) after a median time of 1 and 2 years (n=number of patients entering the time interval).


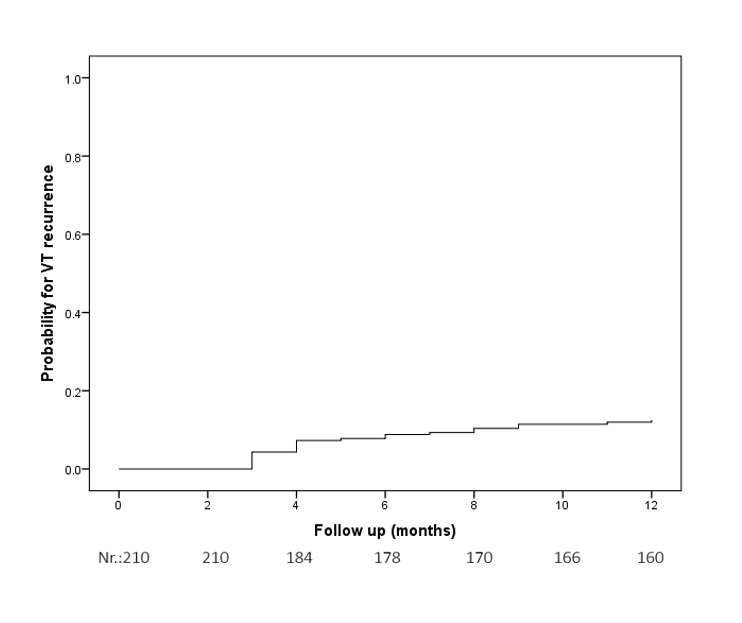


a.


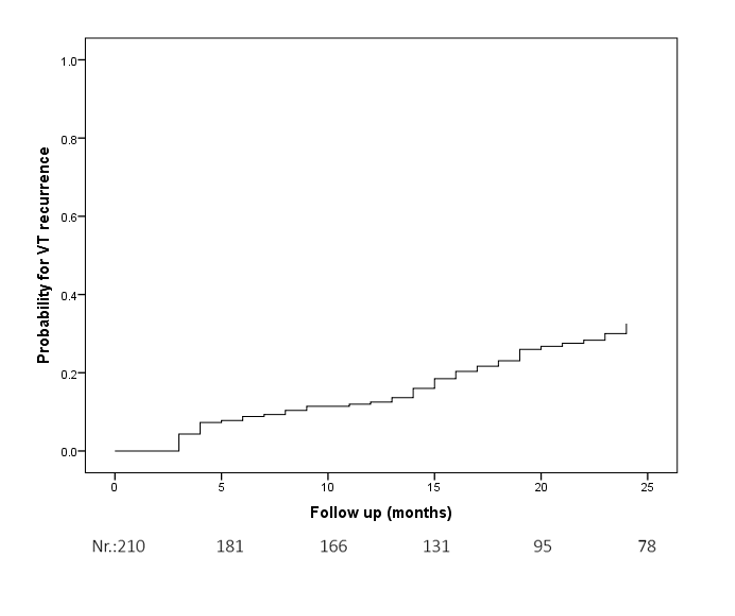


b.


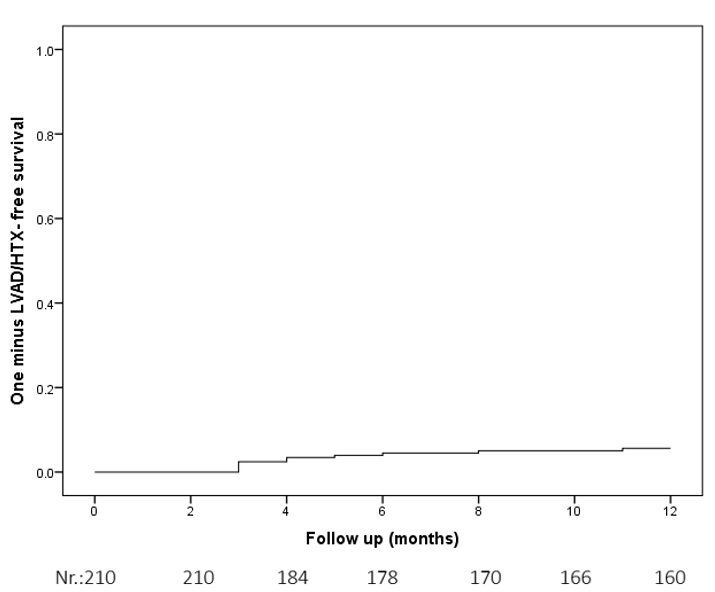


c.


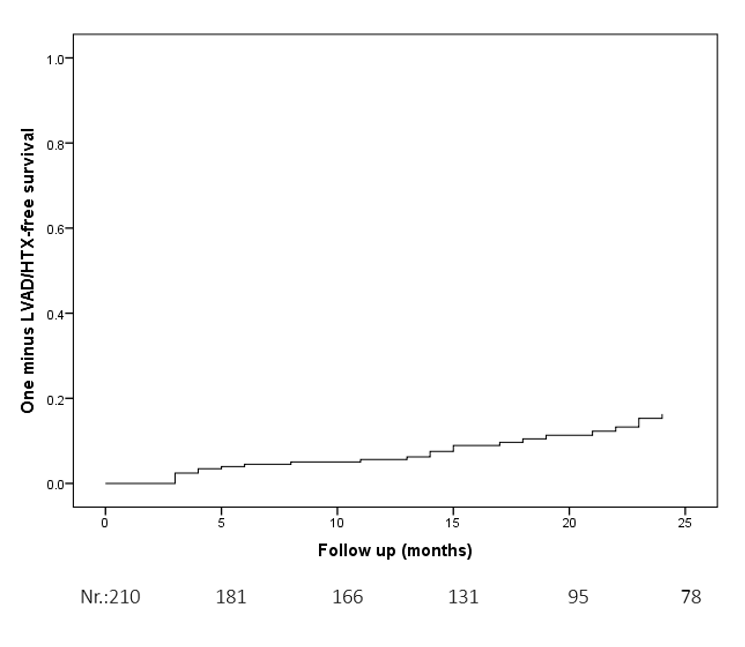


d.
